# The Role of Lactate in Mitochondrial Metabolism of DOX‐Induced Senescent AC16 Cells

**DOI:** 10.1002/cbf.70110

**Published:** 2025-08-12

**Authors:** Rosamaria Militello, Simone Luti, Tania Gamberi, Manuela Leri, Alice Santi, Matteo Becatti, Alessio Pellegrino, Pietro Amedeo Modesti, Alessandra Modesti

**Affiliations:** ^1^ Department of Biomedical, Experimental and Clinical Sciences “Mario Serio” University of Florence Florence Italy; ^2^ Department of Experimental and Clinical Medicine University of Florence Florence Italy

**Keywords:** DOX‐induced senescent AC16 cells, metabolomics, mitochondrial dysfunction, proteomics

## Abstract

Senescent cells accumulate with age in organ and tissue causing the decline of functionality and various pathological conditions including cardiovascular disease. Regular exercise induces continuous exposure to lactate that contribute to adaptive process through mitochondrial biogenesis and improve of metabolic process. Lactate accumulation during exercise also appears to be associated with exercise‐induced mitochondrial adaptation. Improvement of mitochondria function through lactate exposure could be a tool to prevent cardiomyocytes senescence and cardiac aging. The aim of the following article is to investigate the role of lactate in Doxorubicin‐induced senescent AC16 human cardiomyocytes cell mitochondrial metabolism. We assessed the metabolic behaviour in senescent cardiomyocytes after chronic lactate exposure and provided a discussion of the effect of this metabolite in regulating mitochondrial physiology during cardiac aging.

## Summary

Plasma lactate levels increase in elderly and during training in athletes alterations in signalling pathways may influence lactate metabolism and mitochondrial disorders leading to lactate accumulation and subsequently affecting cardiac function. The results obtained highlight its potential impact on mitochondrial metabolism in senescent cardiomyocytes.

## 1 Introduction

Ageing is a lifelong process that occurs in all living organism. Senescent cells accumulate with age in organ and tissue causing the decline of functionality [1] and various pathological conditions including neurodegenerative and cardiovascular disease [2]. Cellular senescence refers to a stable cell cycle arrest in response to intrinsic and extrinsic factors [3]. Despite their growth is quiescent, senescent cells are metabolically active in fact, increased glucose uptake and glycolysis is one of the major metabolic changes [4] beyond a distinct phenotype [3].

Ageing is a complex biological phenomenon that represents in fact the major risk factor for developing cardiovascular diseases [5]. Heart is an organ with high‐energy demand since it must contract incessantly to guarantee oxygen supply to all organs in the body. Its metabolism is highly flexible and can vary widely depending on energy substrate availability: while in healthy heart, under resting condition, fatty acids are the major fuel substrate [6], failing heart increase the use of glucose for ATP production [7]. Cardiomyocytes represent ∼30%–40% of the total cellular population of heart [8] and different from the non‐myocytes, mitochondria content in cardiomyocytes is up to 70% [9]. Mitochondria play a fundamental role in the survival and function of cardiomyocytes [10]. They not only providing more than 90% of the ATP necessary for contraction, but also regulate intracellular Ca^2+^ signalling, lipid metabolism, production of reactive oxygen species (ROS), and apoptosis [11]. In senescent cardiomyocytes, the fission–fusion progress of mitochondria is imbalanced and the function is declined. Mitochondrial dysfunction and alteration of metabolism pattern are the keys feature of cardiomyocyte senescence [12, 13] that in turn contributes to cardiac aging, dysfunction, and failure [9].

Understanding the interplay between senescence, cardiomyocyte metabolism, and mitochondrial health is important to identify strategies for intervening in age‐related cardiac complications. In a previous article, we investigated the effect of growing on lactate instead of glucose in human cardiomyocyte cell line AC16 assessing their viability, cell cycle activity, oxidative stress, and metabolism by a proteomic and metabolomic approach. We found that the exposure for 72 h to lactate promotes cardiomyocytes cytoskeletal remodelling, affects cardiomyocytes metabolism, increase mitochondrial respiration and succinate production [14]. Lactate, a by‐product of glycolysis, is now recognized as a key molecule that connects cellular metabolism with the regulation of cellular activity [15]. Lactate is a myokine that appears to be a mediator of exercise adaptations and health benefits [16], as much to be termed “lactormone” [17]. Lactate, shuttling intracellular and intercellular between producer (driver) and consumer (recipient) cells, acts as a signalling molecule, a major energy source and gluconeogenic precursor [18].

Regular exercise induces continuous exposure to lactate that contribute to adaptive process through mitochondrial biogenesis via the activation of peroxisome proliferator‐activated receptor gamma coactivator‐1 alpha (PGC‐1α) and improve of metabolic process [19]. Its accumulation during exercise also appears to be associated with exercise‐induced mitochondrial adaptation [20]. Improvement of mitochondria function through lactate exposure could be a tool to prevent cardiomyocytes senescence and cardiac aging.

The aim of the following article is to investigate the role of lactate in DOX‐induced senescent AC16 cells mitochondrial metabolism. With this in mind, we assessed the metabolic behaviour in senescent cardiomyocytes after chronic lactate exposure and provided a discussion of the effect of this metabolite in regulating mitochondrial physiology during cardiac aging.

## 2 Materials and Methods

### 2.1 Chemicals

Dulbecco's Modified Eagle's Medium (DMEM) high glucose (ECB7501L) and low glucose (ECM007L) was purchased from Euroclone (Milan, Italy). Nutrient Mixture Kaighn's Modification (F‐12K Nut Mix; 21127‐022) and DMEM without d‐glucose and sodium pyruvate (11966–025) were purchased from Gibco (Thermo Fisher Scientific, Waltham, MA, USA). AC16 Human Cardiomyocyte Cell Line, sodium l‐lactate (L7022) and d‐glucose (G7021) were purchased from Merck (Darmstadt, Germany).

### 2.2 Cell Culture

Experiments were performed on Human Cardiomyocyte Cell Line (AC16). AC16 cells derived from non‐proliferating primary cultures of adult ventricular heart tissue. Cells were handling according to manufacturer protocols with minor changes. Briefly, AC16 were thawed and expanded in DMEM high glucose/F‐12K Nut Mix containing 10% FBS (Fetal Bovine Serum; Euroclone, Cat. No ECS5000L) 1X l‐Glutamine (Euroclone, Cat. No. ECB3000D), 100 units/mL penicillin, 100 μg/mL streptomycin (Euroclone, Cat. No ECB3001D) at 37°C and 5% CO2 until they reached confluence. Then Trypsin/EDTA (Euroclone, Cat. No. ECB3052D) was used to detach cells that were count and reseed at the density of 12,5 × 10^2^ cells per cm^2^ in DMEM low glucose. All experiments were performed using cells until passage ten.

Senescent cells were obtained treating cells 24 h with 0.1 μM of Doxorubicin as reported by Kastury et al., [21]. Then culture media was replaced with DMEM without d‐glucose, 10% FBS, 1X glutamine and 1X penicillin/streptomycin The concentration of sodium lactate 8 mM is the closest to the physiological concentration after training as reported by Stagemann [22]. The experiments were then carried out in senescent (S) and no‐senescent (C) cells separately grown for 48 h on media with three different carbon sources (glucose 5.5 mM (G); glucose 5.5 mM + lactate 8 mM (GL) and lactate 8 mM (L). Most experiments were performed with cells on 60‐mm dishes. For ATP assay 96‐well plates were used, while 24‐well plates were used for the ROS measurement.

### 2.3 Cell Count

Cells were plated in a 24 well plates and treated as describe before. After 48 h of growth with 8 mM sodium l‐lactate, 5.5 mM d‐glucose or both carbon sources, cells were detached with trypsin, resuspended in media and counted using the Burker's chamber and optical microscope. Three wells per condition were count. The experiment was repeated three times.

### 2.4 Microscope Images

To evaluate cellular morphology, images were taken under an inverted microscope Nikon Eclipse TS2 equipped with a 4 K UHD Multi‐output HDMI Camera.

Actin cytoskeleton was stained as reported by Nolfi et al., [23]. Alexa Fluor™ 488 Phalloidin (Thermo Fisher Scientific; A12379) and DAPI (4′,6‐diamidino‐2‐phenylindole dihydrochloride) (Sigma; D9542) were used to label filamentous actin and nuclei respectively. Images were collected using a Leica TCS SP8 scanning microscope (Leica, Mannheim, Ge) equipped with 63×, 1.4–0.6 NA, oil, HCX Plan APO lens.

### 2.5 Apoptosis Cellular Assay

Dead cell apoptosis kit with annexin V FITC and propidium iodide (PI) for flow cytometry (Invitrogen; V13242) was used to differentiate live, dead, and apoptotic cells.

Experiment was carried out according to the manufacture's instruction. Briefly, cells were harvested and washed in cold PBS. Then the supernatants were discarded, and cells resuspended in 100 μL of 1X annexin‐binding buffer and 2,5 μL of FITC Annexin V. Cells were incubated at room temperature for 15 min. After the incubation period, 150 μL of 1X annexin‐binding buffer and 1 μL of PI were added to samples, mixed gently and cells were analysed by FACSCantoII flow cytometer (BD Biosciences, New Jersey, USA). Cells were gated and plotted on dot plots showing the distribution of annexin V‐positive cells (early apoptotic cells), annexin V/propidium iodide‐positive cells (late apoptotic cells), propidium iodide‐positive cells (necrotic cells).

### 2.6 Mitochondrial Transmembrane Potential by Tetramethylrhodamine Ethyl Ester (TMRE)

Mitochondrial membrane potential was measured using the fluorescence probe TMRE. Briefly, AC16 cells were detached with trypsin, after the centrifugation cells were resuspended and incubated for 20 min with 500 μL of PBS with 50 nM of TMRE. Cells were centrifuged for remove the excess probe and resuspended in 200 μL of PBS. Probe intensity was measured using BD Fluorescence‐activated cell sorting (FACS) Canto II (BD Biosciences, Becton Dickinson Europe Holdings SAS ‐ Francia).

### 2.7 Oxygen Consumption Rate (OCR) Analysis

Cells were detached, count and 80000 cells were suspended in 1 mL of medium. The cell suspension was transferred to an airtight thermostatic chamber maintained at 37°C. Cardiomyocytes oxygen consumption, measured by using a Clark‐type O_2_ electrode (Oxygraph Hansatech) for 10 min, has been achieved by taking the rate of oxygen consumption (nmol/min/mL) as an index of respiratory ability. Carbonylcyanide‐3‐chlorophenylhydrazone (CCCP) a protonophore, which causes uncoupling of proton gradient, was then added to cells in the airtight thermostatic chamber and the rate of oxygen consumption was then measured for 3 min.

### 2.8 Mitochondria Labelling

MitoTracker Red dyes M22425 (ThermoFisher Scientific) stain active mitochondria in live cells for mitochondrial labelling. As reported by manufacturer MitoTracker Red FM (Cat. No. M22425) dye is sequestered by functioning mitochondria. However, cells stained with this dye retain their fluorescent staining patterns even if mitochondrial function is disrupted or if cells are subjected to fixation and permeabilization. This property make it useful morphology marker that, once bound, is independent of mitochondrial function [24].

In particular cells were plated on slides; live cells were incubated for 30 min with 500 nM MitoTracker probe. The mitochondrial staining dyes passively diffuse across the plasma membrane and accumulate in active mitochondria. Nuclei were stained with Hoechst 33342 probe 1 μg/mL incubated for 10 min. After the incubation period, cells were washed with PBS and fixed with 4% paraformaldehyde for 10 min. After PBS washing, slides were mounted fluoromounting media. Multicolor images were collected using a Leica TCS SP8 scanning microscope (Leica, Mannheim, Ge) equipped with 63×, 1.4–0.6 NA, oil, HCX Plan APO lens. The images were acquired using the Leica LAS‐AF image acquisition software and analysed the fluorescence intensity and size particles using the FiJi software [25]. Mitochondria smaller than 0,4 µm^2^ were subtracted from the analysis.

To evaluate mitochondrial fragmentation, six randomly chosen fields were selected, and their size/shape was measured on skeletonize imaged obtained with ImageJ plugin (https://imagej.net/plugins/analyze-skeleton/) accordingly with workflow for pre‐processing and analysis of images [26].

### 2.9 ATP Assay

The EnzyLightTM ADP/ATP Ratio Assay Kit (BioAssay Systems, Hayward, USA) provides a simple and direct procedure for measuring ATP levels in cells. Cells were plated in 96‐well plate and experiment was carried out according to the manufacture's instruction. Briefly, the working reagent lyses cells to release ATP. In the presence of luciferase, ATP immediately reacts with the substrate d‐luciferin to produce light. The light intensity is a direct measure of the intracellular ATP concentration. The values of luminescence were normalized for the number of cells.

### 2.10 Intracellular Reactive Oxygen Species (ROS)

The level of intracellular ROS was measured in control and senescent cells 24 and 48 h after the treatments through the probe dichlorofluorescin diacetate (DCFH‐DA) 2500 μM in Dimethyl sulfoxide (DMSO) as reported by Galli et al. [27].

The assay was performed on 24 well plate; medium was replaced with phosphate buffered saline (PBS) containing the probe at the final concentration of 10 μM.

Fluorescence was measured every 10 min with a Sinergy H1 plate reader (BioTek Winooski, Vermont, USA) at excitation/emission wavelengths of 485/538 nm, for a total of 1 h.

Fluorescence intensity was normalized by measuring the total protein content with the BCA assay. Cells were then lysed in 200 μL of water with 0,03% of sodium dodecyl sulphate (SDS).

### 2.11 Western blot Analysis

AC16 were lysed in cold RIPA buffer (150 mM NaCl, 100 mM NaF, 2 mM EGTA, 50 mM Tris HCl pH 7.5, 5 mM orthovanadate, 1% triton, 0.1% Sodium dodecyl sulphate (SDS), 0.1% protease inhibitor cocktail, 0.1% phosphatase inhibitors) for 10 min. Then samples were sonicated 4 times for 5 s at 30% intensity and then clarified by a centrifugation for 10 min at 14 000 rpm.

The total amount of protein was assessed by Bradford assay and an equal amount of protein for each sample was added to 4 × Laemmli buffer (0.5 M TrisHCl pH 6.8, 10% SDS, 20% glycerol, β‐mercaptoethanol, and 0.1% bromophenol 45 blue). Samples, except for those used to see OXPHOS, were boiled for 5 min and separated on 12% SDS/PAGE and transferred onto a PVDF membrane using the Trans‐ Blot Turbo Transfer System (Bio‐Rad Laboratories, Hercules, CA, USA). PVDF was probed with primary antibody p21 (PTM Bio, Chicago, USA), MCT4 (GeneTex, Irvine, CA) PGC1α, LDHA, LDHB, PHD, Citrate synthase, Succinate dehydrogenase subunit A, Actin (Santa Cruz, Texas, USA); OXPHOS (Invitrogen, Massachusetts, USA) diluted 1:1000 in 2% milk, and then incubated overnight at 4°C. After incubation with horseradish peroxidase (HRP)‐ conjugated anti‐mouse IgG or antirabbit IgG (1:5000) (Santa Cruz Biotechnology, Texas, USA), immune complexes were detected with the enhanced chemiluminescence (ECL) detection system (GE Healthcare, Chicago, IL, United States) and by Amersham Imager 600 (GE Healthcare).

Blot was subjected to densitometric analysis using the ImageJ 1.53 program. The intensity of the immunostained bands was normalized with the total protein intensities measured by Coomassie brilliant blue R‐250 from the same PVDF membrane blot as previously reported [28].

### 2.12 Extracellular and Intracellular pH and Lactate Measurements

To provide a quick check for the pH in cell lysates and culture media we used the Phenol red pH indicator as described in Ferguson et al. [29].

The measurement of intracellular (cellular lysate) and extracellular (culture medium) lactate was performed using the K‐LACTATE assay (Magazyme). The assay consists of two reactions. During the first reaction lactate is oxidized to pyruvate by NAD+ and l‐LDH. Since l‐LDH can catalyse the reaction in both directions until equilibrium is reached, a second reaction that traps the produced pyruvate is necessary. In the second reaction d‐glutamate and d‐GPT convert the pyruvate to 2‐oxoglutarate. The assay measures the increase in NADH concentration which occurs during the reaction and is linear over the range of 0,3–30 µg of l‐lactic acid per assay. The absorbance was read at 340 nm before and 10 min after adding l‐LDH. Data obtained from the lactate and pyruvate assays were normalized to the total amount of protein. To quantify total protein, cells were lysed adding 100 µL of a solution of water 0.3% SDS directly in multiwell, stirred for 1 h, and analysed with BCA assay.

### 2.13 Statistical Analysis

Shapiro‐wilk test was performed to assess the normality of data, all of them pass the test.

A two‐way ANOVA was performed for the factors: senescence and cellular culture media (glucose, glucose and lactate, lactate). Test was followed by Tukey's multiple comparison test that were performed using GraphPad Prism 8. Data are presented as means ± standard deviation (SD) from at least three experiments. *p* < 0.05 were considered statistically significant (*p < 0.05; **p < 0.01; ***p < 0.001; ****p < 0.0001).

## 3 Results

### 3.1 DOX‐Induced Senescent AC16 Cells Growth in Different Carbon Sources

AC16 human cardiomyocyte cell line was selected as cellular model and in our experimental condition, was preferred to primary human cardiomyocytes. Primary cultures could be maintained only for few weeks, undergo morphological and functional changes over time and yield a heterogeneous population of cells [30]. AC16 cells derived from non‐proliferating primary cultures of adult ventricular heart tissue made proliferating using a novel method that consists of the fusion of primary cells with SV40 transformed, uridine auxotroph human fibroblasts, devoid of mitochondrial DNA. The presence of the combination of transcription factors in addition to muscle‐specific markers indicate the presence of a cardiac transcription program in these cells, as well the expression of myogenic markers and a fully functional respiratory chain show that this cell line has retained the nuclear DNA and the mitochondrial DNA of the primary cardiomyocytes [31].

AC16 senescence was caused by doxorubicin treatment. This treatment is a model of induced cell senescence [32], and regarding AC16 this treatment is reported by Kastury et al., [21]. In particular, senescent cells were obtained treating cells with 0.1 μM doxorubicin (DOX) for 24 h in DMEM low‐glucose, on the contrary non senescent cells were grown in DMEM low‐glucose for 24 h without doxorubicin. Senescent (S) and no‐senescent (C) cells were then separately grown on media with three different carbon sources (glucose 5.5 mM (SG and CG); glucose 5.5 mM + lactate 8 mM (SGL and CGL) and lactate 8 mM (SL and CL)) [33]. Several authors reported that it is not possible to use a fixed value for the optimal plasma lactate concentration because it may vary between individuals from 1.4 mM to 7.5 mM. Our idea started from several results obtained in our previous studies, where we verified, in plasma from athletes at rest but during the training period, a high‐level concentration of lactate compared to ordinary people of the same age [28]. The achieved senescence was verified by the upregulation of marker cyclin‐dependent kinase inhibitor 1 A (p21) [33] through western blot analysis.

**Figure 1:**
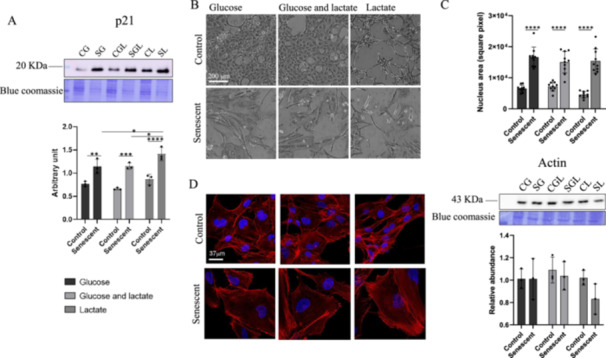
A Representative Immunoblot image and histogram of p21. Senescent cells (S) were grown in glucose 5.5 mM SG; glucose 5.5 mM + lactate 8 mM SGL and lactate 8 mM SL; no‐senescent cells (C) were grown in glucose 5.5 mM CG; glucose 5.5 mM + lactate 8 mM CGL and lactate 8 mM CL. Normalization of immunoblot was performed on Coomassie‐stained PVDF membrane. Histogram displays the results as mean values and SD of three independent biological experiments (*p < 0.05; **p < 0.01; ***p < 0.001; ****p < 0.0001). (B) Optical microscope images; all panels are the same magnification, scale bars for all images: 200 µm. (C) Nucleus area measurement performed using Image J. (D) Actin cytoskeleton by a phalloidin (red)/DAPI (blue) staining and representative immunoblot image and histogram of Actin. Histogram displays the results as mean values and SD of three independent biological experiments (****p < 0.0001). Scale bars for all images: 37 µm.

As reported in Figure 1A cells treated with doxorubicin express higher levels of p21 compared to their respective controls in detail: SG shows a 47% increase (*p* = 0.008) compared to CG, SGL shows an increase of 72.36% (*p* = 0.0005) in comparison to its respective control; SL shows an increase of 64.18% (*p* < 0.0001) in comparison to its control (CL). SL shows also higher levels of p21 in comparison to SG 30.63% (*p* = 0.011) and SGL 27.19% (*p* = 0.0208).

### 3.2 DOX‐Induced Senescent AC16 Cells Morphology

In our model, senescent cells show a peculiar morphology in comparison to the controls. These differences are evident on optical microscopy as reported in Figure 1B, in which all senescent cells appear bigger than the controls. Figure 1C, shows an increase in nucleus area of senescent cells in comparison to respective controls regardless of the carbon sources in media.

In particular, the nuclei's area of SG is 154.7% larger than its control (CG); SGL shows an increase of 109% in comparison to CGL and in SL cells, the increase is 237.6% compared to its control (CL).

Although the change in morphology and size of senescent cells is clearly evident, this does not correspond to a change in the expression level of actin as shown in the Figure 1D in which, using confocal microscopy and immunoblot, we investigated actin cytoskeleton structure.

### 3.3 Cellular Growth and Viability Assay in AC16 Grown in Different Carbon Sources

Senescent cells were counted 48 h after the growth in different carbon sources.

**Figure 2:**
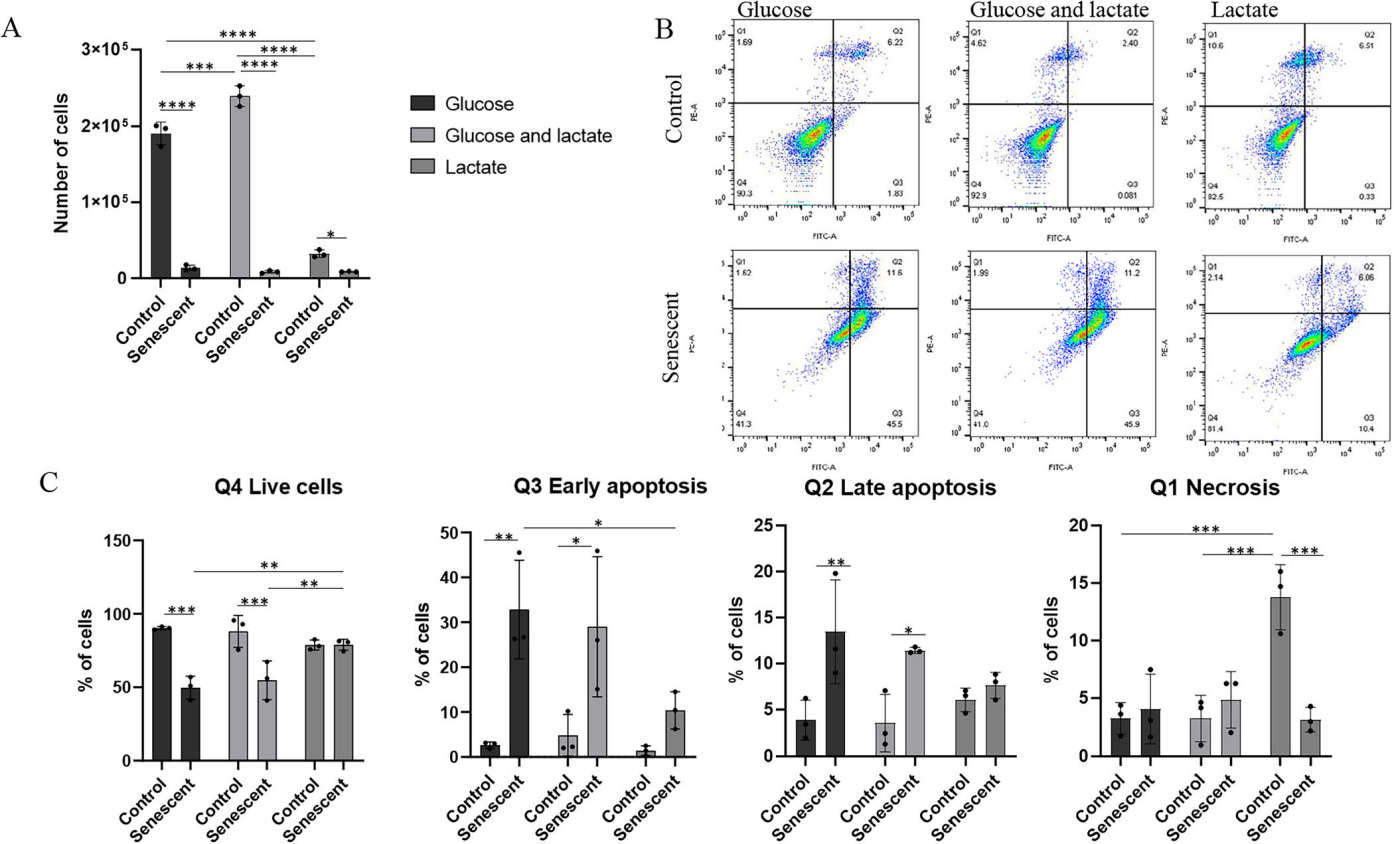
Cellular growth and viability assay. (A) Number of cells 48 h after the growth in different carbon sources. (B) Representative image of flow cytometry analysis of annexin V/propidium iodide‐stained. (C) Histograms that represent the percentage of live, apoptotic, and necrotic cells obtained by flow cytometric analysis. Results are reported as mean of three independent experiments (*p < 0.05; **p < 0.01; ***p < 0.001; ****p < 0.0001).

As reported in Figure 2A, SG shows a decrease in cells number of about 13.4 times in comparison to its control (*p* < 0.0001) and SGL shows a decrease in cells number of 22.6 times compared to CGL (*p* < 0.0001). The number of SL cells pointed out a smaller difference compared to its controls (CL) (3.5 less; *p* = 0.027). Moreover, as reported in Figure 2A, control cells grown in lactate (CL), show a clear reduction in growth state compared to controls grown in glucose (CG 5.8 times) and glucose + lactate (CGL 7.4 times).

To assess the state of cell viability, we carried out a flow cytometric analysis using Annexin V FITC and propidium iodide. The results are shown in Figure 2B. Regarding the percentage of live cells (Q4 panel), there is no difference between control and senescent cells grown in lactate (78.77% for CL and 78.9% for SL), conversely, the percentage of living cells in senescent groups growing in glucose (SG) (49.7%) and glucose+lactate (SGL) (54.77%) are lower than their controls (CG 90.26%, *p* = 0.0001; CGL 88%, *p* = 0.0008 respectively). Moreover, senescent cells growing only in lactate have the highest percentage of live cells compared to the other two senescent conditions (SG, *p* = 0.002; SGL, *p* = 0.009).

As reported in Figure 2C, cells with the single label of annexin V are in early apoptosis (Q3 panel). The results point out that all the control groups have a very low percentage of early apoptotic cells while this percentage increase in senescent groups. Indeed, the percentage of cells increase as following:

1‐ For controls grown in glucose (CG) from 2.65% to 32.83% for senescent cells grown in glucose SG (*p* = 0.0028);
2‐ For controls grown in glucose and lactate (CGL)from 5.19% to 29% for senescent cells SGL (*p* = 0.0147).
3‐ For controls growth in lactate this difference is less evident and the percentage increase is from 1.40% for CL to 10.38% for senescent cells SL. Senescent cells in lactate have the lowest percentage of early apoptotic cells compared to other senescent groups and this difference was significant compared to SG (*p* = 0.0211).

Cells labelled with both probes were the ones in late apoptosis (Q2 panel), there is no difference between control and senescent cells in lactate (CL 6.06% and SL 7.63%) conversely in glucose the percentages change from 3.84% of control to 13.47% for senescent (*p* = 0.0045) and from 3.56% of CGL to 11.43% for SGL (*p* = 0.017).

The staining with only PI indicates cells in necrosis (Q1 panel). PI marks only cells that have a broken cellular membrane. The percentages of necrotic cells did not change between control and senescent cells growing with glucose (3.22% for CG, 4.05% for SG) and glucose and lactate (3.22% for CGL, 4.84% for SGL). On the contrary control cells growing in lactate show the highest percentage of necrotic cells (13.77%) compared to the respective senescent (SL 3.12%, *p* = 0.003) and to other control groups (*p* = 0.003).

### 3.4 DOX‐Induced Senescent AC16 Cells Mitochondrial Metabolism

Since mitochondrial dysfunction is one of the hallmarks of cellular senescent [34], we decide to deeply investigate the effects of lactate on mitochondrial metabolism in our cellular model. Lactate acts as a mitochondrial messenger to stimulate oxidative phosphorylation [35] and promotes transcriptional changes that induce mitochondrial biogenesis [36].

#### 3.4.1 Measurement of Mitochondrial Transmembrane Potential

**Figure 3:**
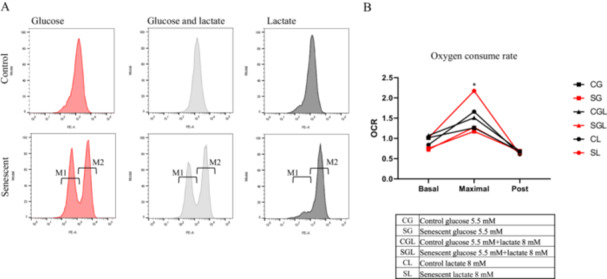
Mitochondrial function. (A) Representative image of flow cytometry analysis of TMRE stained, on the X‐axis the relative fluorescence, on the Y‐axis the number of events. Senescent cells histograms show two peaks: one with low fluorescence intensity (M1) and another with high intensity (M2). (B) Graph that represent the Oxygen consume rate at basal level, after the addition of CCCP (maximal) and at the end of its effect (post). Results are reported as mean of three independent experiments (*p < 0.05).

Mitochondrial transmembrane potential was measured by tetramethylrhodamine ethylesterpercholate (TMRE) which is a fluorescence lipophilic dye that accumulates in active mitochondria. As reported in Figure 3A a single peak with a relative fluorescence of 0.61 for CG, 0.60 for CGL, and 0.64 for CL is evident. Instead, senescent cells histograms show two peaks: one with low fluorescence intensity (M1) and another with high intensity (M2). M1 represents mitochondria with low or absent TMRE incorporation typically found in cells under apoptosis or necrosis, while M2 represents functional mitochondria [37]. Although the relative fluorescence value of both peaks shows no differences between senescent cells groups, the percentage of events that constitute each peak change significantly as reported in Table 1. In fact, while the percentages of M1 peak events in SG and SGL were 39.1% and 39.7% respectively, in SL were only 11.5%. SL therefore has a higher percentage of functional mitochondria; the percentage of events in M2 is in fact 88% against 60.6% of SG and 60.1% of SGL.

**Table 1:** Percentages of events in M1 and M2 peaks in senescent groups. All measurements were performed in triplicate and are reported as percentage mean ± SD.

Cellular treatment	M1	M2
SG	39.1 ± 10.3	60.6 ± 10.4
SGL	39.7 ± 5.1	60.1 ± 5.4
SL	11.5 ± 2.1	88 ± 2

To confirm that senescent cells grown in lactate shown better mitochondrial activity, we measured oxygen consume rate at basal condition and after the addition of an uncoupling agent like Carbonylcyanide‐3‐chlorophenylhydrazone (CCCP). The addition of CCCP induces uncoupling of proton gradient in the inner mitochondrial membrane causing the rapidly increase of oxygen consume rate [38].

As reported in Figure 3B, in basal condition there are no statistically significant differences in oxygen consumption among groups [38].

As shown in the figure, the greater response to CCCP is observed in senescent cells grown in lactate (SL) in which the oxygen consume rate increase of 86.3% (*p* = 0.0139) compared to SG and 70.3% (*p* = 0.0312) compared to SGL. This confirms that in SL mitochondria are initially coupled and became uncoupled in response to CCCP while in SG and SGL there are already uncoupled mitochondria and therefore CCCP have a reduced action on these samples accordingly with TMRE analysis (M1 peaks).

#### 3.4.2 Measurement of Mitochondria Number and Size

**Figure 4:**
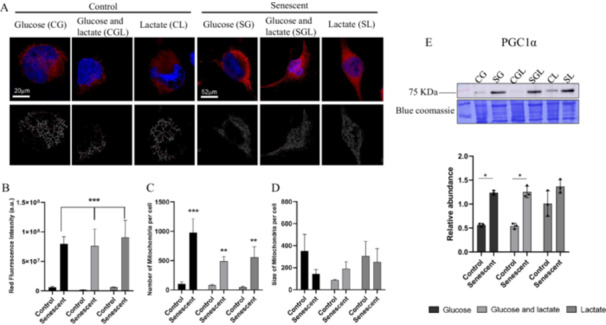
Mitochondria number and size. (A) Representative confocal images of AC16 mitochondria. Organelles were stained with Mitotracker Red (red signal) and Nuclei with Hoechst 33342 (blue signal). The respective reproductions of mitochondrial skeleton were reported in the bottom panel (black and grey). Scale bars for control images are 20 µm, scale bars for senescent 52 µm. Mitochondria skeletons were reported in the bottom panel and the quantification of their (B) intensity, (C) number and (D) size calculated by ImageJ were reported in the histogram graphs as mean values and SEM of three independent experiments (**p < 0.01; ***p < 0.001). (E) Representative immunoblot image and histogram of PGC1α. Normalization of immunoblot was performed on Coomassie‐stained PVDF membrane (*p < 0.05).

Mitochondrial number and size were determined using the mitochondria‐specific dye, MitoTracker Red, images are reported in Figure 4A. Figure 4B shows fluorescence intensity increase in all senescent groups compared to their controls (*p* < 0.001) indicating a higher number of mitochondria per cell compared to their controls. In particular, as reported in Figure 4C, SG shows an increase of 854,6% in mitochondria compared to CG (*p* < 0.001), SGL shows a 516.3% of increase in comparison to CGL (*p* = 0.009) and the increase in mitochondria number for SL is of 1147% in respect to CL. The size of mitochondria was also calculated but no significant differences emerged among groups as reported in Figure 4D.

The expression of Peroxisome proliferator‐activated receptor‐gamma coactivator 1‐alpha (PGC‐1alpha), a transcriptional coactivator [39] that regulates the mitochondrial biogenesis [40] was evaluated by western blot as reported in Figure 4E. The immunoblot analysis shows an increase of 125% (*p* = 0.0329) in SG in comparison to CG and an increase of 140% in SGL in comparison to CGL (*p* = 0.0229). This difference between controls and senescence is not evident in cells growth on lactate.

#### 3.4.3 The Oxidative Phosphorylation (OXPHOS) System

To evaluate mitochondrial activity, we investigated the OXPHOS system using western blot.

**Figure 5:**
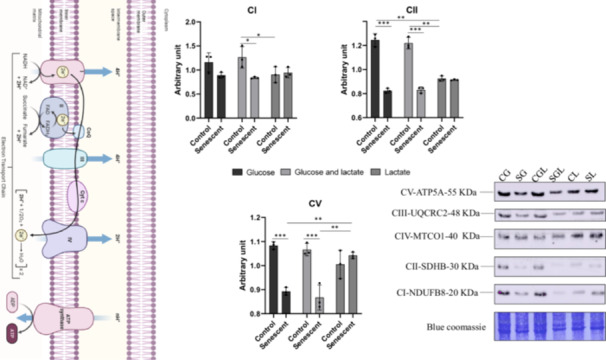
The oxidative phosphorylation system (OXPHOS). Representative immunoblot image of all complex of OXPHOS. Senescent (S) and no‐senescent (C) cells separately grown on media with three different carbon sources (glucose 5.5 mM (G); glucose 5.5 mM + lactate 8 mM (GL) and lactate 8 mM (L)). Normalization of immunoblot was performed on Coomassie‐stained PVDF membrane. Histograms display the statistically significant differences identified in CI, CII, and CV. Results are reported as mean values and SD of three independent biological experiments (*p < 0.05; **p < 0.01; ***p < 0.001).

As reported in Figure 5, in senescent cells grown in glucose+lactate (SGL) complex I decreased of 58% (*p* = 0.016) compared to its corresponding control (CGL). Moreover, in CL the complex shows a decrease of 53, 5% (*p* = 0.023) compared to CGL.

The expression of complex II (SDHB) shows a significant decrease of 33.1% (*p* = 0.0004) in senescent cells grown in glucose (SG) compared to its control (CG) and the reduction in expression value for SGL is about 31.4% (*p* = 0.0008) compared to CG. These differences are not evident between senescent and control cells growing in lactate. In fact, the reduction in expression level of complex II in CL cells is about 25.3% (*p* = 0.005) compared to CG and 24.3% (*p* = 0.008) compared to CGL.

Complex III and Complex IV show no statistically significant difference in expression levels (Supporting Figure 1).

Complex V decreases of about 22.3% (*p* = 0.0007) in SG compared to CG and of 23.3% (*p* = 0.0006) in SGL compared to CGL. In cells growth with lactate as only carbon source, the Complex V expression level shows no statistical differences in control and senescent cells. Level of Complex V in SL is higher of about 17.6% (*p* = 0.0058) compared to SG and of 21.3% (*p* = 0.0015) than SGL.

#### 3.4.4 Measurement of ATP Levels

**Figure 6:**
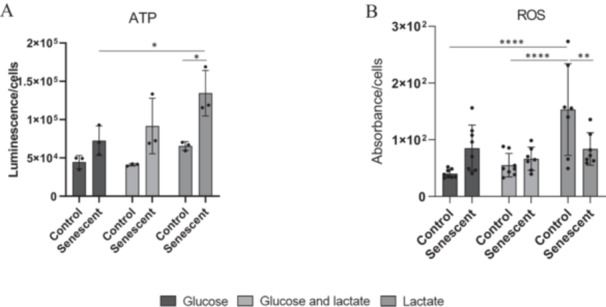
ATP levels and ROS measurement. (A) Measurement of ATP level. (B) Measurement of ROS levels by using the 2′‐7′‐Dichlorodihydrofluorescein diacetate assay (DCFH‐DA). Results are reported as mean values and SD of three independent biological experiments (*p < 0.05; **p < 0.01; ***p < 0.001; ****p < 0.0001).

To evaluate the metabolic state of cells we analysed the ATP levels. Results are reported in Figure 6A in which is evident that senescent cells show higher ATP levels than their respective controls. In particular, we observe an increase of 105,17% (*p* = 0.0172) in senescent cells grown on lactate compared to its control (CL), and an increase of 85,42% (*p* = 0.034) compared to senescent cells but grown on glucose (SG).

### 3.5 ROS Measurements

The measurement of intracellular ROS by DCFDA revealed that control cells growing in lactate show higher level of ROS in comparison to CG (+282.10%; *p* < 0.0001) CGL (+178.27%; *p* < 0.0001) and SL (+82.93%; *p* = 0.0067). These differences are not found among senescent groups. Results were reported in Figure 6B.

### 3.6 Enzymes of the Oxidative Metabolism

**Figure 7:**
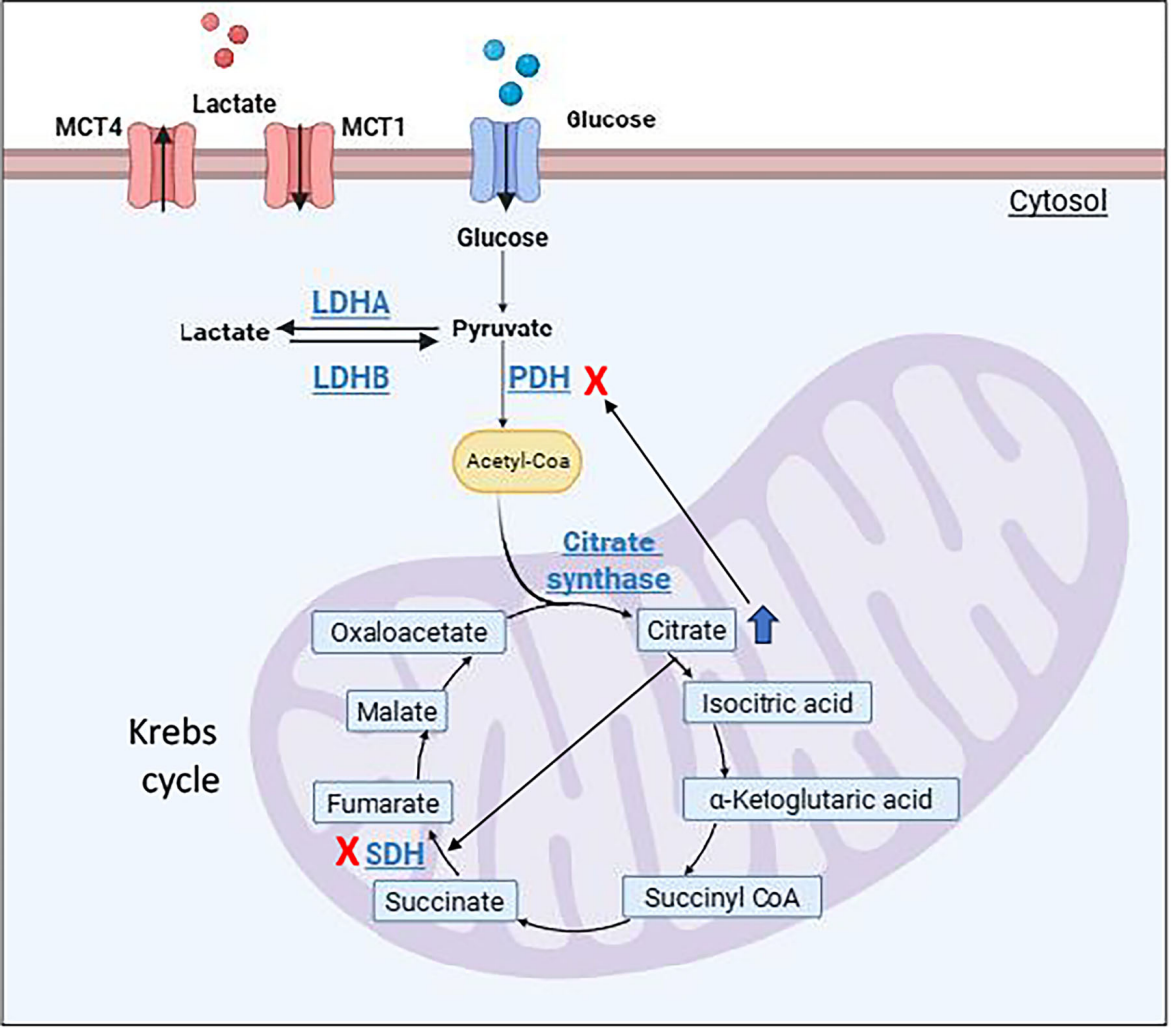
Graphic summary of the pathways involving the studied enzymes.

To deeply investigate the mitochondrial functionality, we analysed the level of enzymes involved in the oxidative metabolism. The enzymes lactate dehydrogenase (LDH), pyruvate dehydrogenase (PDH), succinate dehydrogenase (SDH), and citrate synthase are involved in the process of energy generation through the Krebs cycle and play a crucial role in regulating the flux of metabolites and energy into the mitochondria. They are interconnected:PDH provides the acetyl‐CoA required for citrate synthesis by the enzyme citrate synthase, the citrate produced can inhibit PDH and SDH, regulating the flux through the TCA cycle and SDH is involved in the subsequent steps of the TCA cycle, downstream of citrate synthase. We decided to evaluate their expression level in our cellular model. Figure 7 summarizes the pathways in which these enzymes are involved.

#### 3.6.1 Pyruvate Dehydrogenase (PDH)

**Figure 8:**
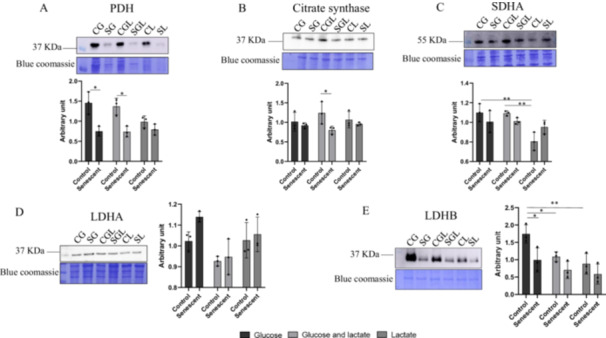
Representative immunoblot image and histogram of (A) Pyruvate dehydrogenase (PDH); (B) Citrate synthase; (C) Succinate dehydrogenase subunit A (SDHA); (D) Lactate dehydrogenase A (LDHA); (E) Lactate dehydrogenase B (LDHB). SG and CG: glucose 5.5 mM; SGL and CGL: glucose 5.5 mM + lactate 8 mM; SL and CL: lactate 8 mM. Normalization of immunoblot was performed on Coomassie‐stained PVDF membrane. Histograms display the results as mean values and SD of three independent biological experiments (*p < 0.05; **p < 0.01).

We evaluated the expression level of the pyruvate dehydrogenase (PDH) that catalyses the oxidative decarboxylation of pyruvate to acetyl‐coenzyme A. PDH decreases of 97.5% (*p* = 0.0125) in senescent cells grown in glucose (SG) and of 95.3% (*p* = 0.0193) in senescent cells grown in glucose+lactate (SGL) in comparison with their respective controls (CG and CGL) but these differences are not evident in control (CL and SL) and senescent cells grown on lactate as reported in Figure 8A.

#### 3.6.2 Citrate Synthase

Citrate synthase catalyses the rate‐limiting for TCA cycle.

In Figure 8 B SGL shows a decrease in citrate synthase expression level in comparison to its control (−54.88%; *p* = 0.0368). This difference is not evident between senescent and control cells growing in the other conditions (SG and CG; SGL and CGL).

#### 3.6.3 Succinate Dehydrogenase Subunit A

The SDH complex (4 subunits: A, B, C, and D) is located on the inner membrane of the mitochondria and participates in both the citric acid cycle and the respiratory chain. The SDH complex catalyses the oxidation of succinate to fumarate. Electrons removed from succinate transfer to SDHA, transfer across SDHB to the SDHC/SDHD subunits on the hydrophobic end of the complex anchored in the mitochondrial membrane. In OXPHOS analysis (Figure 5), we investigated the level of the subunit SDHB as a part of electron transport chain. Herein we analysed SDHA level as a part of the TCA cycle. SDHA expression remains unchanged among senescent groups but varied among controls as reported in Figure 8C. In particular, cells grown on lactate have lower levels in comparison both with glucose (−36.52%; *p* = 0.0017) and glucose and lactate (−35.78%; *p* = 0.0020).

#### 3.6.4 Lactate Dehydrogenase A and B

The immunoblot analysis for LDHA (Figure 8D) shows no statistical differences among groups, on the contrary, LDHB (Figure 8E) shows a decrease level in expression in SG in comparison to CG (−75.5%; *p* = 0.0352). In cells grown on lactate there is no difference between control and senescent in line with the data on SDHB. Among control groups and in cells grown in lactate, LDHB expression level decrease. In fact, in comparison to CG, the expression level of LDHB in CGL is 60.3% lower; while in CL it is 97.3% lower.

### 3.7 Measurement of Extracellular and Intracellular pH, Lactate and Monocarboxylate Transporter

**Figure 9:**
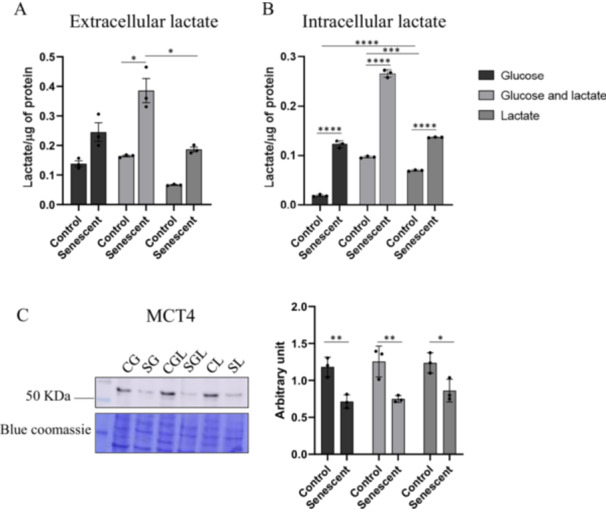
(A) Levels of extracellular lactate normalized for the quantity of proteins; (B) Levels of intracellular lactate normalized for the quantity of proteins. (C) Representative immunoblot image and histogram of monocarboxylate transporter 4 (MCT4): SG and CG: glucose 5.5 mM; SGL and CGL: glucose 5.5 mM + lactate 8 mM; SL and CL: lactate 8 mM. Normalization of immunoblot was performed on Coomassie‐stained PVDF membrane Histograms display the results as mean values and SD of three independent biological experiments (*p < 0.05; **p < 0.01; ***p < 0.001; ****p < 0.0001).

To ensure that the effects we observed were not due to a change in pH, we evaluated this parameter in the culture media and in the cell lysates in the various conditions and we did not detect variations in this parameter (data not shown). Moreover to verify that the findings we observed did not reflect the different levels of lactate uptake between controls and senescent cells we measured the levels of extracellular and intracellular lactate as shown in Figure 9A,B respectively. The levels of extracellular lactate in senescent cells grown in glucose+lactate (SGL) are 145% (*p* = 0.0167) higher compared to its control (CGL) and 111.7% (*p* = 0.0285) higher compared to senescent cells grown on lactate (SL). We observed an increase of intracellular lactate level (Figure 9B) in all senescent groups in comparison to their controls (*p* < 0.0001). In particular, in senescent cells grown in glucose (SG) there is an increase of 544.72% compared to their controls (CG), in senescent cells grown in glucose+lactate (SGL) the increase of 174.9% compared to the corresponding controls (CGL). In senescent cells grown in lactate as carbon source (SL), the increase is lower compared to other two growth conditions and this value is 97.14% compared to their control cells (CL). Statistically significant differences are evident also among controls groups. The level of intracellular lactate in CL decrease in comparison to CGL (−38.74%, *p* = 0.0001) and increase in comparison to CG (+265.2%, *p* < 0.0001). To confirm these differences, we verify the expression level of monocarboxylate transporter 4 (MCT4). The immunoblot analysis shows a decrease level in expression in all senescent cells in comparison to their controls as shown in Figure 9C. In particular senescent cells growth in glucose shows a decrease of 65.50% (*p* = 0.004) compared to CG cells, senescent cells growth in glucose and lactate shows a decrease of 67,62% (*p* = 0.002) compared to their respective controls and senescent cells growth in lactate shows a decrease of 43,37% (*p* = 0.018) compared to their control cells.

## 4 Discussion

Human cardiac AC16 cells showed increasingly common use in studying human cardiomyocyte biology and disease mechanisms. Although adult cardiomyocytes have already exited the cell cycle, the induction of premature senescence in response to cardiotoxic stimuli such as oxidative stress can lead to many major cardiac pathologies. Therefore, we used AC16 cells and the treatment with doxorubicin (dox) as model senescence and aging in vitro. Our aim was to evaluate the involvement of lactate in dox‐induced senescent AC16 cells. The induction of senescence is often recognized in various cell types using markers such as the cell cycle regulatory protein p21, which is activated in senescent cells as reported by Kastury et al., [21].

In this study, we confirmed the overexpression of p21 in all senescent cells regardless of the carbon sources. Surprisingly, the statistical analysis pointed out that the variation of p21 was determined not only by the treatment with doxorubicin but also by the carbon source being p21 higher in non‐senescent AC16 grown on lactate 8 mM. Since the role of lactate in the last several decades has profoundly changed and it is now recognized as a signalling molecule and in a previous paper we evaluated the effect of lactate on human cardiomyocyte and found changes in several proteins and metabolites linked to cell hypertrophy and cytoskeleton remodelling [14]. With this in mind, we assessed the metabolic behaviour in senescent cardiomyocytes after chronic lactate exposure at the levels found in athletes, and we provided a discussion of the effect of this metabolite in regulating mitochondrial physiology during aging. In addition, we used a medium containing only lactate to understand how cells respond to lactate accumulation. In line with the data presented in literature [2, 41, 42], we observed that all senescent cells have a completely different morphology compared to non‐senescent cells. However, lactate also affected non‐senescent cells that appeared longer and thinner than non‐senescent cells grown on glucose e glucose/lactate. We also found that the number of cells decreased significantly in all senescent cells. In addition, this difference in our experimental model was less evident between senescent and non‐senescent AC16 grown on lactate due to the ability of lactate to slow down the cell growth as we reported in a previous study [41][14]. It is well known that cardiomyocyte senescence can lead to or be caused by oxidative stress, metabolic dysfunction, decreased myocardial contractility, inflammation and fibrosis [9]. Mitochondria are dynamic organelles and in myocardium during senescence mitochondrial fusion decreases and mitochondrial division increases, resulting inactivity of mitochondrial enzymes, and thus impacts myocardial energy metabolism. Furthermore, mitochondrial dysfunction is fundamental in the induction of senescence and the latter is related to the decline of mitochondrial function and to an enhancement of glycolytic metabolism [43].

Therefore, in our senescent cells we analysed the effect of different carbon source on mitochondrial metabolism. In our experiment, we found that dox‐induced senescent AC16 cells grown in 8 mM lactate showed a phenotype that highlighted less mitochondrial damage and better viability compared to media containing glucose. Moreover, we found that lactate as the sole carbon source attenuates mitochondrial dysfunction in senescent AC16 cells. Indeed, our results show that doxorubicin‐induced senescence in AC16 cells grown on glucose and glucose/lactate exhibited an increase in early and late apoptosis and a reduced vitality compared to lactate growth condition. This apoptotic phenotype was also associated with a decrease in mitochondrial membrane potential damage.

On the contrary, lactate significantly inhibits DOX‐induced oxidative stress and mitochondrial dysfunction showing after 48 h of treatment a higher number of viable cells and a high respiratory capacity.

These findings suggest that cardiomyocytes growth in lactate rapidly switch from aerobic fermentation mode to respiration, thus regulating fundamental pathways in the survival mechanism.

To be sure that acidosis was not responsible for some of the results obtained, we found no pH modifications in comparison to the pH values of different media used. Moreover, since the levels of intracellular and extracellular lactate increased in all senescent cells and the expression of the transporter was reduced in all senescent cells regardless of the growth medium, we can conclude that the difference observed in cells grown in lactate are not due to a different uptake of lactate but rather to a different mitochondrial activity.

This metabolic shift from fermentation to respiration undergo when rapidly switching from a medium containing glucose to a medium with only lactate as a carbon source could be responsible for the initial metabolic shock that leads to a clear reduction in growth state compared to cells grown in presence of glucose. On the contrary, cells that after this initial stress continue to grow in the presence of lactate for further 48 h, show a recovery of vitality that does not occur in senescent cells maintained in a medium in which glucose is present. Table 2 reports the main differences between senescent cells growth with lactate and glucose.

**Table 2:** Summary differences between senescent cells growth with lactate (SL) and glucose (SG). ↑ show that the value increases or ↓decreases in SL in comparison to SG. Detailed results are reported in the Results section (*p < 0.05; **p < 0.01).

	SL vs. SG
Cyclin‐dependent kinase inhibitor 1A (p21)	↑*
Live cells by flow cytometric analysis	↑**
Early apoptosis by flow cytometric analysis	↓*
Maximanl oxygen consume rate	↑*
ATP synthase (Complex V)	↑**
ATP level	↑*

The intrinsic significance of this mechanism involving l‐lactate will need to be further investigated, but it suggests a role for this molecule as the main regulator of mitochondria biology during aging. In this perspective, l‐lactate may be a key factor in regulating mitochondrial physiology. Interestingly, recent data reported that intermittent treatment with l‐lactate induced a mild inhibition of the mitochondrial aerobic process through the respiratory chain (mito‐hormesis) [44], causing ROS production, AMPK phosphorylation and activation of mitochondria biogenesis. Moreover, ROS act as mediator of various physiological processes such as cell differentiation and proliferation, cell metabolism, survival, and immune response [45].

Overall, our findings seem to support the idea of lactate as a signalling messenger involved in the regulation of senescent metabolism [43]. However, our knowledge about the mechanisms by which lactate could modulate senescent cardiomyocytes metabolism needs to be further explored and therefore more efforts are needed.

## 5 Conclusion

Energy substrates in heart microenvironment are central regulators of cardiomyocyte senescence since the metabolism switch is a key feature for the homeostasis and senescence of cardiomyocytes. As thus, an interesting point is to investigate how the increase of circulating lactate in physiological conditions such as due to physical activity affects the metabolic pattern of cardiomyocyte undergoing senescence. Further studies are needed to explore how metabolism alternations contribute to cardiomyocyte senescence and cardiac aging.

### 5.1 Limitation

In this study, we compared the results obtained treating senescent AC16 cells growth with 8 mM lactate with those obtained with 5.5 mM glucose and 5.5 mM glucose + 8 mM lactate and we know that this study design is not close to physiological conditions. However, as reported in the manuscript, our idea emerged from the results obtained in our previous study in which human cardiomyocytes grown with lactate as the main carbon source showed oxidative metabolism towards mitochondrial respiration. The cells showed a period of adaptation with partial cell death due to the sudden change of the carbon source from fermentable to oxidative. Furthermore, lactate‐grown cardiomyocytes rapidly switch from aerobic fermentation mode to respiration, thus regulating key pathways in the survival mechanism. With this in mind, we characterize the metabolic profile of lactate in senescent cardiomyocyte cells to detail the metabolic flexibility of the heart during senescence. Due to an incomplete understanding of the biological functions of lactate, its biochemical significance during aging in myocardial metabolism is still underestimated. In our experimental model, we attempted to isolate the effects of lactate alone on senescent cells. In our experimental approach, using a medium containing only lactate can help to better understand the metabolic response of senescent cells following an accumulation of lactate over time. Despite the limitations due to the use of lactate as a sole carbon source, we believe that the results of this study are very promising and yet further experiments will be essential to fully elucidate the complex role of lactate on myocardial metabolism during senescence.

## Author Contributions

**Rosamaria Militello:** writing – original draft, methodology, investigation, data curation, conceptualization. **Simone Luti:** investigation, formal analysis. **Alice Santi:** validation, investigation, formal analysis. **Manuela Leri:** investigation, formal analysis. **Matteo Becatti:** investigation, formal analysis. **Tania Gamberi:** writing – review and editing, resources, investigation, formal analysis. **Alessio Pellegrino:** resources, formal analysis. **Pietro Amedeo Modesti:** writing – review and editing, supervision. **Alessandra Modesti:** writing – review and editing, validation, supervision, resources, funding acquisition, data curation, conceptualization.

## Ethics Statement

We use a commercial cell line (AC16 Human Cardiomyocyte Cell Line from Merk).

## Conflicts of Interest

The authors declare that they have no known competing financial interests or personal relationships that could have appeared to influence the work reported in this paper.

## Supporting information


**Supporting Figure 1:** Histograms display the quantification of complex CIII and CIV of OXPHOS systems reported in Figure 5. Results are reported as mean values and SD of three independent biological experiments.

## Data Availability

The authors confirm that the data supporting the findings of this study are available within the article.
