# Supplementary figures and images for: The Role of Lactate in Mitochondrial Metabolism of DOX‐Induced Senescent AC16 Cells
Source: Cell Biochem Funct. 2025 Aug 12;43(8):e70110. doi: 10.1002/cbf.70110 (PMC12341654; doi:10.1002/cbf.70110)

**CIII**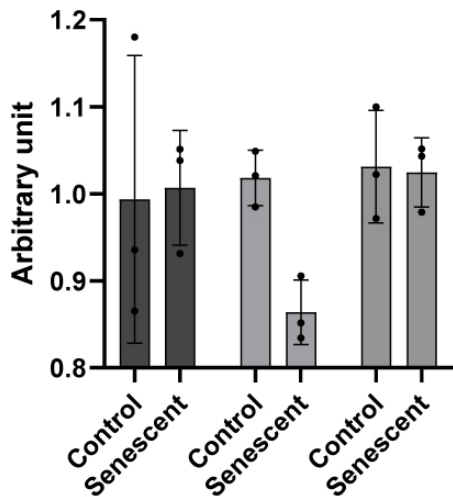**CIV**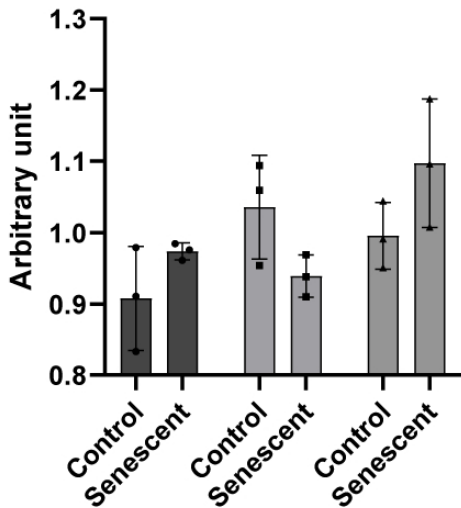

■ Glucose    ■ Glucose and lactate    ■ Lactate

Supplement: Supplementary file 1 — Supporting Figure 1: Histograms display the quantification of complex CIII and CIV of OXPHOS systems reported in Figure 5. Results are reported as mean values and SD of three independent biological experiments. [file CBF-43-e70110-s001.pdf]
